# Double attack strategy for leukemia using a pre-targeting bispecific antibody (CD20 Ab-mPEG scFv) and actively attracting PEGylated liposomal doxorubicin to enhance anti-tumor activity

**DOI:** 10.1186/s12951-020-00752-w

**Published:** 2021-01-09

**Authors:** Kai-Wen Ho, I.-J.u Chen, Yi-An Cheng, Tzu-Yi Liao, En-Shuo Liu, Huei-Jen Chen, Yun-Chi Lu, Yu-Cheng Su, Steve R. Roffler, Bo-Cheng Huang, Hui-Ju Liu, Ming-Yii Huang, Chiao-Yun Chen, Tian-Lu Cheng

**Affiliations:** 1 Graduate Institute of Medicine, College of Medicine, Kaohsiung Medical University, Kaohsiung, Taiwan; 2 Drug Development and Value Creation Research Center, Kaohsiung Medical University, Kaohsiung, Taiwan; 3 Department of Biomedical Science and Environmental Biology, Kaohsiung Medical University, No. 100 Shih-Chuan 1st Road, Kaohsiung, 80708 Taiwan; 4 Department of Medical Research, Kaohsiung Medical University Hospital, Kaohsiung, Taiwan; 5 Department of Biomedical Science and Environmental Biology, Kaohsiung Medical University, Kaohsiung, Taiwan; 6 Institute of Molecular Medicine and Bioengineering, Department of Biological Science and Technology, National Chiao Tung University, Hsin-Chu, Taiwan; 7 Institute of Biomedical Sciences, Academia Sinica, Taipei, Taiwan; 8 Institute of Biomedical Sciences, National Sun Yat-Sen University, Kaohsiung, Taiwan; 9 Department of Radiation Oncology, Kaohsiung Medical University Hospital, Kaohsiung, Taiwan; 10 Department of Radiation Oncology, Faculty of Medicine, College of Medicine, Kaohsiung Medical University, Kaohsiung, Taiwan; 11 Department of Medical Imaging, Kaohsiung Medical University Hospital, Sanmin Dist, No.100, Tzyou 1st Rd, Kaohsiung, Taiwan

**Keywords:** Bispecific antibody (CD20 ab-mPEG scFv), Liquid tumors, Internalization, Pegylated nanoparticle, Specific targeting

## Abstract

**Background:**

Tumor-targeted nanoparticles hold great promise as new tools for therapy of liquid cancers. Furthermore, the therapeutic efficacy of nanoparticles can be improved by enhancing the cancer cellular internalization.

**Methods:**

In this study, we developed a humanized bispecific antibody (BsAbs: CD20 Ab-mPEG scFv) which retains the clinical anti-CD20 whole antibody (Ofatumumab) and is fused with an anti-mPEG single chain antibody (scFv) that can target the systemic liquid tumor cells. This combination achieves the therapeutic function and simultaneously “grabs” Lipo-Dox® (PEGylated liposomal doxorubicin, PLD) to enhance the cellular internalization and anticancer activity of PLD.

**Results:**

We successfully constructed the CD20 Ab-mPEG scFv and proved that CD20 Ab-mPEG scFv can target CD20-expressing Raji cells and simultaneously grab PEGylated liposomal DiD increasing the internalization ability up to 60% in 24 h. We further showed that the combination of CD20 Ab-mPEG scFv and PLD successfully led to a ninefold increase in tumor cytotoxicity (LC_50_: 0.38 nM) compared to the CD20 Ab-DNS scFv and PLD (lC_50_: 3.45 nM) in vitro. Importantly, a combination of CD20 Ab-mPEG scFv and PLD had greater anti-liquid tumor efficacy (*P* = 0.0005) in Raji-bearing mice than CD20 Ab-DNS scFv and PLD.

**Conclusion:**

Our results indicate that this “double-attack” strategy using CD20 Ab-mPEG scFv and PLD can retain the tumor targeting (*first attack*) and confer PLD tumor-selectivity (*second attack*) to enhance PLD internalization and improve therapeutic efficacy in liquid tumors.
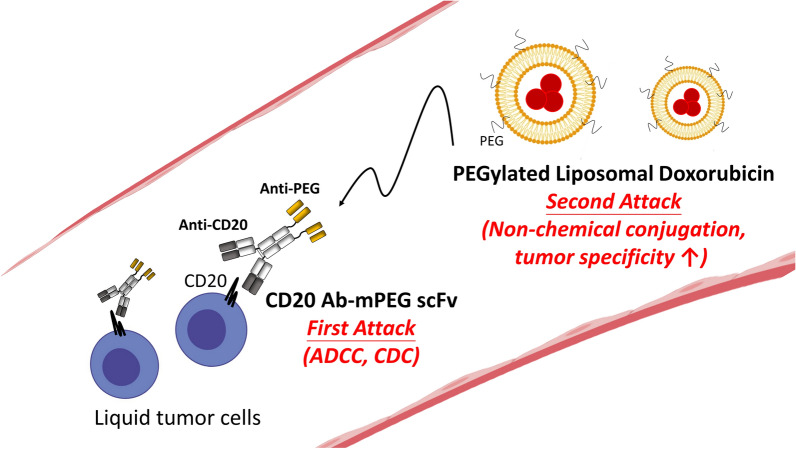

## Background

B-cell lymphoproliferative disorders such as acute lymphoblastic leukemia (ALL) are characterized primarily by an overproduction of immature lymphocytes in the bone marrow which results in the suppression of normal haematopoiesis and infiltration of various extramedullary sites [1]. Rituximab, is a chimeric monoclonal antibody (mAb) that binds to CD20, which is expressed on the membrane of up to 90% of late pre-B and mature B-cells as well as in the majority of B-cell lymphoproliferative disorders [2, 3]. Rituximab can activate complement-dependent cytotoxicity (CDC) via it’s human Fc domain and antibody-dependent cellular cytotoxicity (ADCC) based on the additional contact to Fcγ receptors [2]. In the clinic, the use of doxorubicin hydrochloride combined with Rituximab is an effective therapy for relapsed and refractory leukemia [4]. However, the cardiotoxicity potential due to the low tumor-targeting and multidrug resistance remain issues with doxorubicin hydrochloride usage. Thus, doxorubicin hydrochloride has been used in liposomal doxorubicin formulations like Lipo-Dox® (PEGylated liposomal doxorubicin, PLD) which are less cardiotoxic whilst showing no significant difference with conventional doxorubicin on overall survival of acute lymphoblastic leukemia patients [5, 6]. According to previous studies, nanoparticles may accumulate and be retained in solid tumors through the enhanced permeation and retention (EPR) effect [7, 8]. However, the EPR effect is present in tumors of more than ~ 100 mm^3^ in volume and hampers the use of nanoparticles for targeting small or unvascularized metastases [9]. We hypothesized that the lack of an improvement in outcome of PLD against leukemia may be due to the non-specific targeting cytotoxicity and a reduced EPR effect. Therefore, to retain the anti-tumor effects of the therapeutic antibody, development of a strategy to enhance PLD tumor selectivity against liquid tumors is important.

**Fig. 1:**
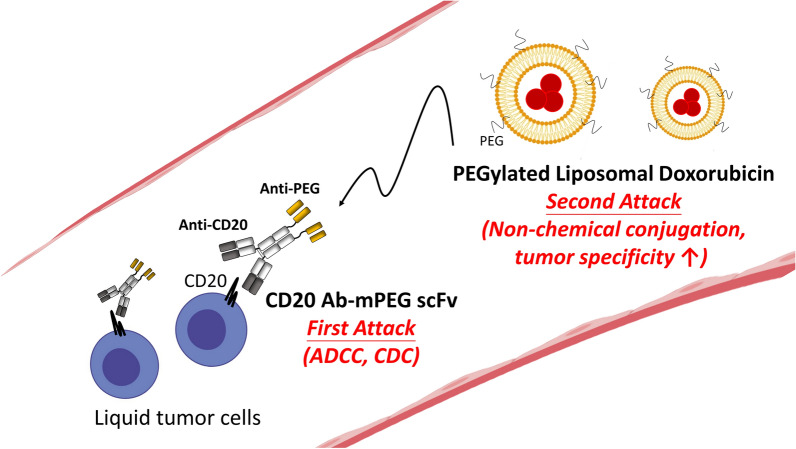
Schematic of the “double-attack” strategy: bispecific antibody combined with PEGylated liposomal doxorubicin. The bispecific antibody (CD20 mAb-mPEG scFv) can simultaneously target the systemic liquid tumor cells, retain the antibody-dependent cellular cytotoxicity (ADCC) and complement-dependent cytotoxicity (CDC) *(first attack)*. After PLD injection, the CD20 Ab-mPEG scFv on the membrane of Raji cells would recognize the mPEG of PLD to cause the “*second attack*” of PLD by enhancing the internalization of PLD and enhance the therapeutic efficacy

In this study, we developed a humanized bispecific antibody (BsAbs: CD20 Ab-mPEG scFv) which retains the clinical anti-CD20 mAb (Ofatumumab) [10] and is fused with humanized single-chain antibodies (scFv) of an anti-mPEG antibody [11] can specific recognize the methyl polyethylene glycol (mPEG) modified on the liposomal doxorubicin. In our double-attack strategy, the first attack is CD20 Ab-mPEG scFv binds to the CD20-expressing Raji cells and trigger the antibody-dependent cellular cytotoxicity and complement-dependent cytotoxicity against Raji cells. After PLD injection, the CD20 Ab-mPEG scFv on the membrane of Raji cells would recognize (or we call it “grab”) the mPEG of PLD to cause the “second attack” of PLD by enhancing the internalization of PLD and cytotoxicity against liquid tumors (Fig. 1a). We examined the bi-specific binding activity of CD20 Ab-mPEG scFv to PLD and CD20-expressing lymphoma cells. Enhanced internalization of PLD into Raji cells mediated by CD20 Ab-mPEG scFv was demonstrated by flow cytometry. In addition, tumor-specific cytotoxicity of CD20 Ab-mPEG scFv combined with PLD was investigated and compared with a control BsAb (CD20 Ab-DNS scFv) combined with PLD and PLD alone in Raji cells. The therapeutic efficacy of CD20 Ab-mPEG scFv combined with PLD was determined and compared with a control BsAb combined with PLD in a Raji-bearing lymphoma model. The CD20 Ab-mPEG scFv provides a powerful “double-attack” method to treat liquid tumors by increasing the tumor-specificity and internalization of PLD under conditions in which the EPR effect is sub-optimal. The CD20 Ab-mPEG scFv is a promising next-generation antibody drug to treat liquid tumors.

## Results

### Characterization of CD20 Ab-mPEG scFv

**Fig. 2:**
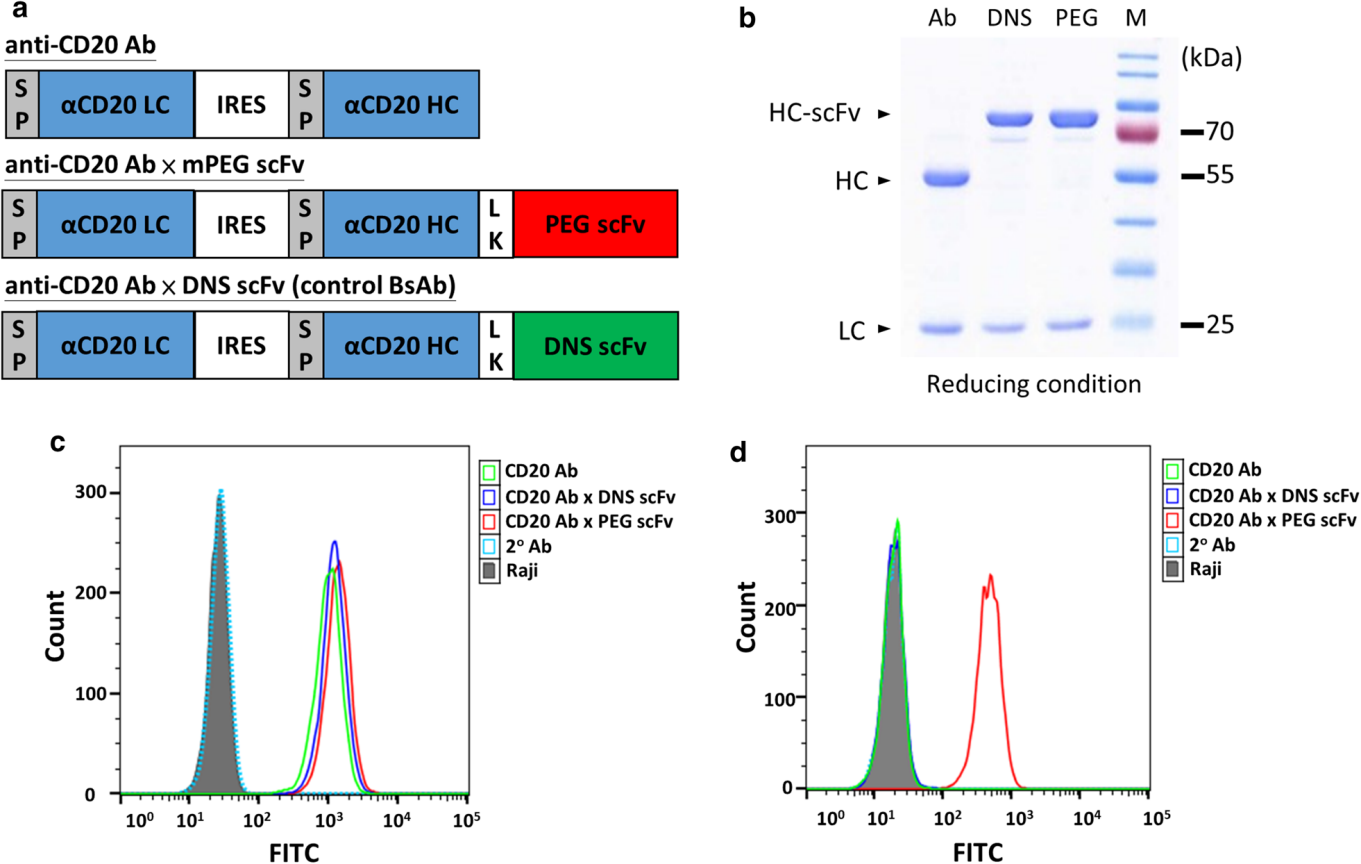
Characterization of CD20 Ab-mPEG scFv, CD20 Ab-DNS scFv and CD20 mAb. **a** The gene constructs of BsAbs are composed of a signal peptide (SP), the anti-CD20 light chain (LC), an internal ribosome entry site (IRES), the anti-CD20 heavy chain (HC), a flexible linker peptide (LK) and an anti-PEG scFv (CD20 Ab-mPEG scFv) or control anti-DNS scFv (CD20 Ab-DNS scFv). **b** The SDS-PAGE of purified CD20 Ab (Ab), CD20 Ab-DNS scFv (DNS) and CD20 Ab-mPEG scFv (PEG) under reducing conditions. M: PageRuler pre-stained protein ladder (Fermentas). **c** The CD20 functions and **d** bispecific binding (CD20 and mPEG) function of BsAbs on Raji (CD20+) cells were detected by anti-Fc-FITC or PEGylated lipo-DiD respectively via flow cytometry

We investigated the bi-specific function of CD20 Ab-mPEG scFv, which was constructed by linking a single-chain variable fragment of a humanized anti-mPEG Ab via a flexible peptide (GGGSGGG) to the C-terminus of Ofatumumab. CD20 Ab-DNS scFv is the negative control compare to the anti-mPEG end of CD20 Ab-mPEG scFv. The anti-DNS scFv end of CD20 Ab-DNS scFv will not recognize the mPEG molecule which modified on the liposomal doxorubicin. Therefore, CD20 Ab-DNS scFv can only target to the CD20-expressing Raji cells to trigger the antibody-dependent cellular cytotoxicity (ADCC) and complement-dependent cytotoxicity (CDC) (first attack). Compare to the CD20 Ab-DNS scFv, CD20 Ab-mPEG scFv not only can target to the CD20-expressing Raji cells to trigger the first attack also recognize the mPEG molecule on the liposomal doxorubicin and induce the internalization to enhance the therapeutic efficacy. (Fig. 2a). CD20 Ab-DNS scFv does not bind mPEG on pegylated liposomal doxorubicin. Therefore, CD20 Ab-DNS scFv can target CD20-expressing cells to trigger antibody-dependent cellular cytotoxicity (ADCC) and complement-dependent cytotoxicity (CDC) *(first attack)*, but cannot bind PLD to induce the second attack. By contrast, CD20 Ab-mPEG scFv can not only trigger the first attack but can also bind mPEG on PLD to target the liposomes into CD20-positive lymphoma cells to enhance the therapeutic efficacy. The recombinant BsAbs were produced in Expi293F cells and purified by Protein A affinity chromatography. The SDS-PAGE analysis shows the purity and expected molecular weight of BsAbs composed of a 76 kDa heavy chain-scFv fragment (HC-scFv) and 25 kDa light chain (LC); the CD20 Ab was composed of a 55 kDa HC fragment and a 25 kDa LC fragment under reducing conditions (Fig. 2b). We further evaluated CD20 binding by incubating CD20-positive Raji cells with 70 nM CD20 Ab-mPEG scFv, CD20 Ab-DNS scFv and CD20 Ab and detecting binding by anti-human Fc Ab-FITC via flow cytometry. The flow intensity, which was similar to CD20 Ab alone indicated that CD20 Ab-mPEG scFv can target CD20-expressing Raji cells as well as the original CD20 Ab (Fig. 2c). Next, the bi-specific binding activity of CD20 Ab-mPEG scFv was examined by incubating PEGylated liposomal DiD after addition of the BsAbs and measuring the bound fluorescence intensity by flow cytometry. Figure 2d shows increased fluorescence intensity of Raji cells treated with CD20 Ab-mPEG scFv and PEGylated liposomal DiD indicating that only the CD20 Ab-mPEG scFv can specifically target PEGylated liposomal DiD to CD20-positive Raji cells. We conclude that CD20 Ab-mPEG scFv can retain the antibody targeting and mediate selective delivery of PEGylated liposomes, thus displaying bi-specific binding function.

### Pre-targeting of CD20 Ab-mPEG scFv can enhance the cellular internalization of PEGylated liposomes.

**Fig. 3:**
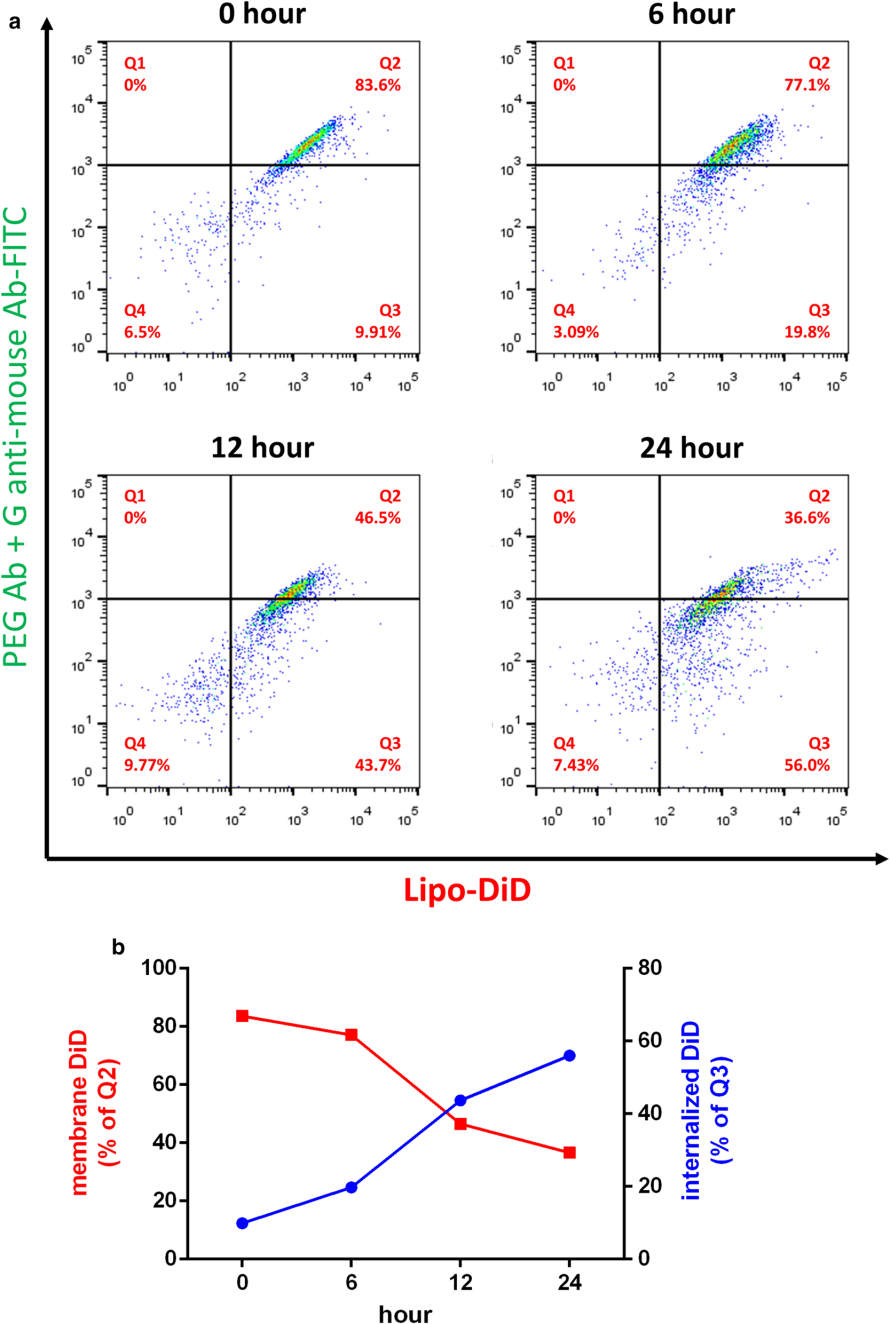
The internalization and PEGylated liposome DiD uptake of CD20 Ab-mPEG scFv in Raji cells. **a** The internalization of CD20 Ab-mPEG scFv combined with Lipo-DiD into Raji cells at different times. The presence of Lipo-DiD on the surface of Raji cells was measured via flow cytometry using a mouse anti-PEG backbone antibody. **b** Quantification of the mean fluorescence of DiD on the surface of Raji cells (red) and DiD uptake efficiency (blue)

The endocytosis of liposomes is important for cancer therapy. We investigated how pre-targeting of CD20 Ab-mPEG scFv contributes to the cellular internalization of PEGylated liposome to enhance cellular cytotoxicity against CD20-positive lymphoma cells. We incubated Raji cells with CD20 Ab-mPEG scFv, CD20 Ab-DNS scFv or CD20 Ab for 1 h and then stained them with PEGylated liposomal DiD (lipo-DiD) for 1 h, 6 h, 12 h and 24 h at 37 °C. Lipo-DiD on the surface of Raji cells was detected by staining with mouse anti-PEG antibody (6.3 antibody) [12] and then staining with anti-mouse Fc Ab-FITC. We detected the two signals, lipo-DiD (red fluorescence) and FITC (green fluorescence) by flow cytometry. The red lipo-DiD signal shows the total liposomes associated with the cells whereas a decreasing green fluorescence signal corresponds to uptake of lipo-DiD since the liposomes are no longer assesible to staining with anti-PEG antibody. Figure 3a shows that green fluorescence (Q2) decreased from 83.6% to 36.6% in time dependent manner, but the total Lipo-DiD signal (Q2 + Q3) did not change (ranging from 92.6% to 93.51%) from 0 to 24 h. These results indicate that the CD20 Ab-mPEG scFv enhances internalization of lipo-DiD in Raji cells. The membrane Lipo-DiD decreased (Q2) and up to 56% of bound Lipo-DiD was internalized into cells within 24 h (Fig. 3b). Moreover, lipo-DiD alone or combined with CD20 Ab-DNS scFv (control group) did not produce detectable green fluorescence intensity, consistent with lack of liposome targeting to the cells. Therefore we conclude that only CD20 Ab-mPEG scFv can enhance cell uptake of liposomes via the anti-mPEG scFv portion of the BsAb.

### Pre-targeting of CD20 Ab-mPEG scFv enhances cytotoxicity of PLD to Raji cells.

**Fig. 4:**
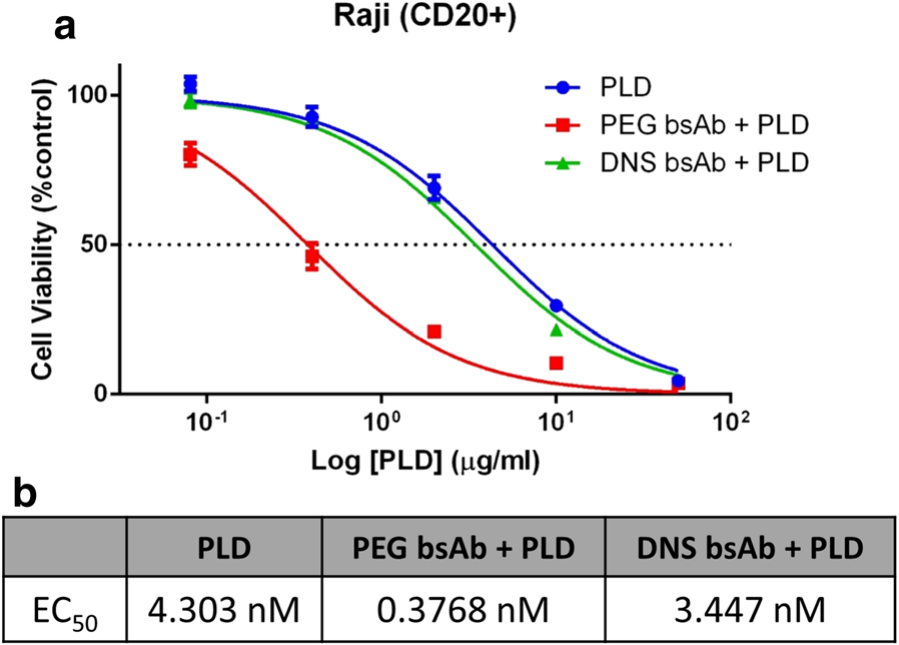
CD20 Ab-mPEG scFv enhances PLD anti-proliferative activity. **a** Raji cells were incubated with CD20 Ab-mPEG scFv (red squares) or CD20 Ab-DNS scFv (green triangles) for 20 min followed by addition of serial dilutions of PEGylated liposomal doxorubicin (PLD) in triplicate for 3 h. The cells were also incubated with culture medium for 20 min followed by addition of serial dilutions of PLD (blue circles). Cell viability was measured with ATPlite Luminescence Assay System after 72 h of incubation. **b** The half maximal effective concentration (EC_50_) values of Raji cells treated with CD20 Ab-mPEG scFv plus PLD, CD20 Ab-DNS scFv plus PLD or PLD alone were analyzed. Data are shown as mean ± s.d. PEG BsAb, CD20 Ab-mPEG scFv; DNS BsAb, CD20 Ab-DNS scFv; PLD, PEGylated liposomal doxorubicin

We next investigated whether CD20 Ab-mPEG scFv could enhance the cytotoxicity of PLD against Raji cells. We first incubated the Raji cells for 20 min with CD20 Ab-mPEG scFv or CD20 Ab-DNS scFv for specific binding and then added graded concentrations of PLD for 3 h at 37 °C. The cells were refreshed with new medium and cultured for 72 h to allow time for the doxorubicin to kill the cancer cells. We then assessed the proliferation of the remaining cells by ATPlite Luminescence Assay System. As shown in Fig. 4a, CD20 Ab-mPEG scFv combined with PLD was more cytotoxic than the control groups. The EC_50_ value of CD20 Ab-mPEG scFv combined with PLD (EC_50_ value = 0.38 nM) was approximately ninefold lower than that for CD20 Ab-DNS scFv combined with PLD (EC_50_ value = 3.5 nM), and 11-fold lower than PLD alone (EC_50_ value = 4.3 nM) as shown in Fig. 4b. Therefore, we conclude that CD20 Ab-mPEG scFv can confer tumor selectivity and increase the cytotoxicity of PLD to CD20-positive Raji cells.

### Pharmacokinetic characterizations of CD20 Ab-mPEG scFv

**Fig. 5:**
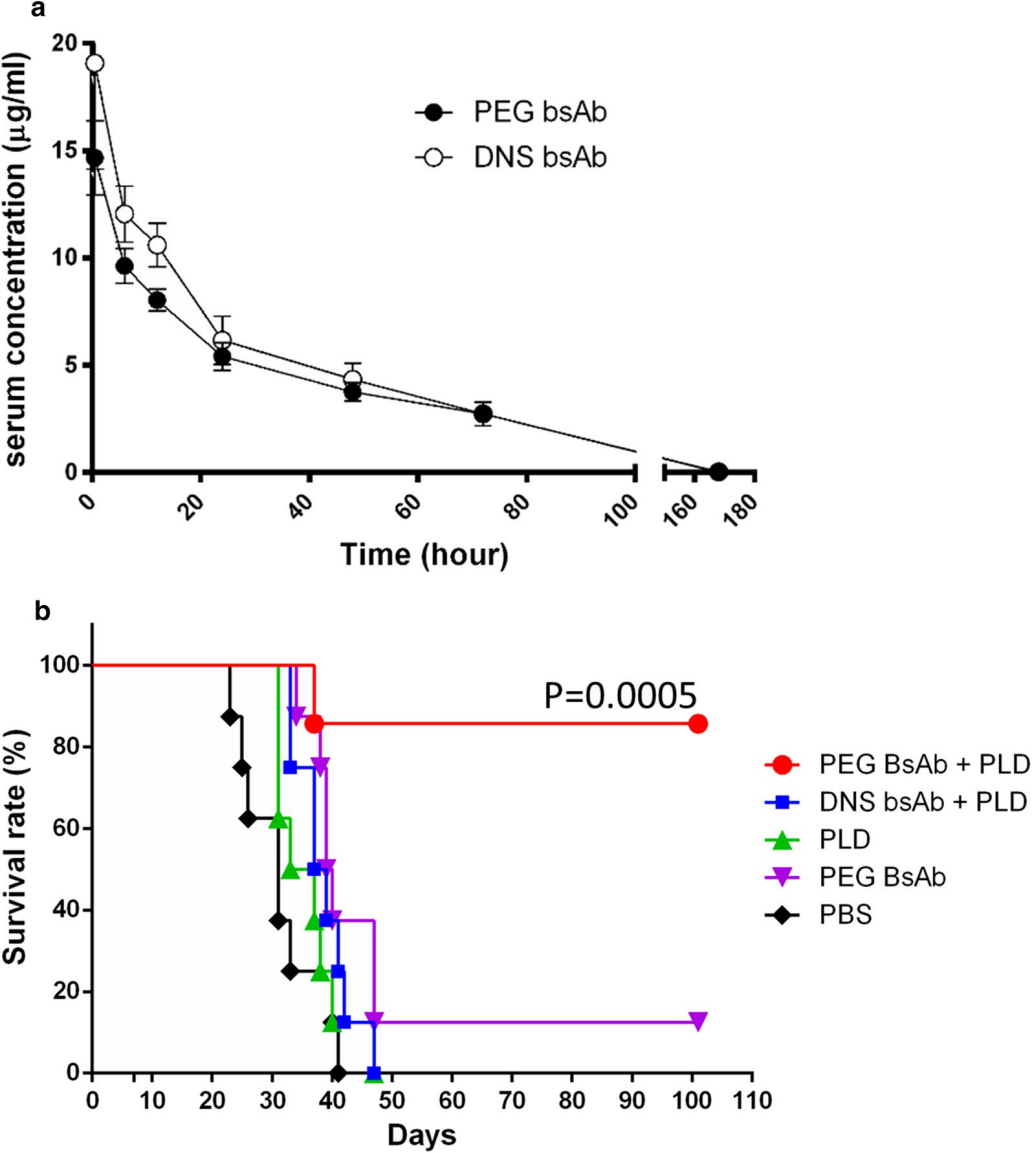
Pharmacokinetics and therapeutic efficiency of BsAbs plus PLD. **a** BALB/c mice were intravenously injected with 1 mg kg^−1^ PEG BsAb (black circles) or DNS BsAb (white circles). Mean plasma concentrations of the BsAbs were measured by sandwich ELISA (n = 3 mice). Data are shown as mean ± SD. n = 3. **b** Groups of eight SCID mice bearing Raji cells were intravenously injected with 1 mg kg^−1^ PEG BsAb plus 2 mg kg^−1^ PLD (blue circles), 1 mg kg^−1^ DNS BsAb plus 2 mg kg^−1^ PLD (red squares), 1 mg kg^−1^ PLD (green triangles), 1 mg kg^−1^ PEG BsAb (purple triangles) or PBS (yellow diamonds). Results are presented as means (n = 8). PEG BsAb, CD20 Ab-mPEG scFv; DNS BsAb, CD20 Ab-DNS scFv; PLD, PEGylated liposomal doxorubicin. Data are shown as mean ± SD. n = 8

To investigate the pharmacokinetics of the bispecific antibodies, 1 mg/kg CD20 Ab-mPEG scFv or CD20 Ab-DNS scFv were injected intravenously into BALB/c mice (n = 3), then serum samples were collected at various times and the concentrations of bispecific antibodies were determined by sandwich ELISA. Herceptin was used as a standard antibody. The half-life (t_1/2_) of CD20 Ab-mPEG scFv was 14.2 h and control BsAb CD20 Ab-DNS scFv was 12.2 h. To minimize the possibility that excess free CD20 Ab-mPEG scFv might clear subsequently injected PLD, we chose 72 h when 85% of the BsAbs was cleared from the circulation as the optimal time for injection of PLD in therapy experiments (Fig. 5a).

### Pre-targeting of CD20 Ab-mPEG scFv increases the therapeutic efficacy of PLD in vivo

To attain a model mimicking systemic tumors, Raji (CD20+) cells were transplanted intravenously into female SCID mice via the tail vein. After 7 days, the mice were randomly administered injections of PBS, CD20 Ab-mPEG scFv or CD20 Ab-DNS scFv. The PLD were injected 72 h after BsAbs administration. Mice were sacrificed at the onset of paralysis or when the body weight dropped below 80% of the initial weight; otherwise, the mice were maintained until 100 days and considered long-term survivors. The group treated with CD20 Ab-mPEG scFv combined with PLD had significantly longer survival time than control groups injected with PBS (*P* = 0.015) or CD20 Ab-DNS scFv combined with PLD (*P* < 0.05) (Fig. 5b). The results indicate that the combination of CD20 Ab-mPEG scFv and PLD can enhance the therapeutic efficacy of PLD in a lymphoma xenograft model.

## Discussion

In this report we provide a novel “double attack” strategy against liquid tumors by using CD20 Ab-mPEG scFv and PEGylated liposomal doxorubicin (PLD). CD20 Ab-mPEG scFv increased the cellular internalization of PLD from 36.6% to 83.6% after 24 h, and enhanced the cellular cytotoxicity by 11-fold as compared with PLD alone in vitro (Fig. 3). Furthermore, a combination of CD20 Ab-mPEG scFv and PLD showed a superior therapeutic effect against circulating Raji cells in mice. Importantly, this double-attack strategy can be adapted for use with any kind of therapeutic antibodies to target different tumor antigens (e.g., CD19, EGFR, HER2, etc.) to become second generation antibody drugs. In addition, the anti-PEG scFv can be combined with diverse PEGylated nanocarriers like liposomes containing different active pharmaceutical ingredients such as paclitaxel (PCX) and irinotecan [13–15]. Similar to PEGylated liposome formulations, polymeric micelles have also been considered to be a promising platform and could be combined with our “double-attack” strategy for cancer therapy [16]. We believe the combination of CD20 Ab-mPEG scFv and PEGylated nanoparticles can be effectively used in the clinic to treat liquid tumors such as B-cell lymphoma and become the next generation antibody drugs.

To confer liposomes with active cancer cell targeting in the circulation is important [17]. Several strategies have been used to chemically conjugate antibodies to liposomes to enhance specificity against liquid cancers. Cong Wu and colleagues chemically conjugated liposomal adriamycin (ADR) with Fab fragments of rituximab and showed that CD20-targeted liposome can specifically target to the tumor site and significantly prolong the survival of tumor-bearing mice [18]. Sun and colleagues conjugated liposomal daunorubicin with CD123/CD33 antibody by thiolation, and attained 1.8-fold higher cellular uptake and 1.53-fold (*P* < 0.001) greater cytotoxicity against acute myeloid leukemia (AML) cells [19]. Strop et al. used microbial transglutaminase that catalyzed bond formation to conjugate an amine-containing drug onto an engineered glutamine on the antibody [20]. However, chemical conjugation caused liposome heterogeneity as most functional groups (amino, carboxyl, thiol groups) are abundant in targeting ligands and lead to limitations in clinical applications for targeted liposomes [20, 21]. Here, our CD20 Ab-mPEG scFv provides the specificity of CD20 to target the liquid tumor and induces the ADCC and CDC effects during the “first attack”. The “second attack” occurs when naïve PLDs are injected and actively bind to the mPEG scFv of the CD20 Ab-mPEG scFv to enhance cellular cytotoxicity against liquid tumor cells. The “double-attack” significantly increases the survival rate of Raji-bearing mice in vivo (*P* = 0.0005) in the absence of an EPR effect. Our strategy overcomes the disadvantages of chemical conjugation and confers the naïve PLD tumor specificity by the mPEG scFv of CD20 Ab-mPEG scFv under non-chemical conjugation of PLD.

The endocytosis ability of liposomes is essential for cancer therapeutic efficacy. Previous studies have revealed the importance of internalization to increase the nanoparticle cytotoxicity efficacy against liquid tumors [22, 23]. Sapra provided evidence showing that using internalizing anti-CD19-immunoliposomes compared with non-internalizing anti-CD20-immunoliposomes significantly improved the therapeutic outcome, prolonging the lifespan of Namalwa cell-bearing mice (*P* < 0.001) [24]. Chuang and colleagues also designed endocytic and nonendocytic receptors by replacing the ligand binding domain of the low-density lipoprotein receptor (LDLR) and a truncated LDLR (ΔLDLR) lacking the NPXY signal motif for endocytosis with αPEG antibodies and stably expressed the receptors on the surface of HCC36 cells. After PLD treatment, HCC36/αPEG-LDLR tumors were significantly suppressed at day 63 as compared with HCC36/αPEG-ΔLDLR tumors in vivo. These results indicate that targeting endocytic receptors can indeed enhance the therapeutic efficacy of PLD [25]. Our CD20 Ab-mPEG scFv first targets the liquid tumor cells and induces ADCC and CDC effects, then the BsAbs remain on the liquid tumor cell membranes until crosslinked with the mPEG part of PLD. There are around 4500 mPEG-DSPEs on a 100 nm liposome [26, 27] and many PEGs on the liposome act like a “Hedgehog” or multiple epitopes which can trigger endocytosis after the PLD has attached mPEG scFv of BsAb [28]. In liquid tumors, CD20 Ab-mPEG scFv combined with PLD enhanced the internalization rate from 10.6% to 60.5% via receptor-mediated internalization (Fig. 3b) and lead to 11-fold greater cytotoxicity in comparison with ordinary combination therapy in vitro*.* Therefore, CD20 Ab-mPEG scFv can increase the internalization ability of PLD to enhance the liquid tumor cytotoxicity.

It is essential to develop a flexible and changeable BsAbs platform in response to resistance of leukemia. Mechanisms of leukemia resistance in vivo are not clear. CD20 loss after treatment with rituximab has been reported that results in a CD20 negative phenotype and resistance to rituximab [29, 30]. Therefore, being able to quickly change the therapeutic target such as CD19 [31] or CD22 [32] is important for the leukemia resistant treatment. For instance, CD19 which is expressed on B-lineage cells becomes a replaceable leukemia target for immunotherapy and has lately been advanced into clinical trials such as anti-CD19 antibody denintuzumab and anti-CD19/CD3 BiTE antibody blinatumumab [33]. Another strategy to deal with leukemia resistance is third generation anti-CD19 CAR-T cells (CTL019 T cells) [34], which provide patients with up to 2 years longer survival against acute lymphoblastic leukemia (ALL) [35]. However, multiple trials have also reported patient relapses due to CD19-negative malignant B cells after anti-CD19 CAR-T cell treatment. In order to combat the resistance of CD19-negative cells, CD19/CD20 CAR-T cells have been used in a clinical trial (NCT04007029) to treat patients with recurrent or refractory B-cell lymphoma. This illustrates that it is important to generate “flexible” treatment strategies to overcome leukemia resistance. Our CD20 Ab-mPEG scFv strategy provides an “adaptable” option (clinical mAb × mPEG) for leukemia-resistant treatment as the CD20-end of the BsAb can be replaced by any kind of anti-leukemia tumor marker Ab after CD20 resistance. Another type of drug resistance occurs when leukemia cells express drug resistant proteins, MDR1 and MRP1, which act as drug efflux pumps, which can mediate doxorubicin resistance [36, 37]. The anti-mPEG scFv of BsAbs can actively “grab” any kind of nanocarrier that is modified with PEG such as PEGylated polymeric micelles [38], Irinotecan [39] or other PEGylated liposomal drugs [40–42]. Therefore, the double-attack strategy of CD20 Ab-mPEG scFv provides adaptable and wide ranging options for combating liquid tumor resistance by swapping in and out different leukemia tumor markers (anti-tumor end) or combining any kind of PEGylated drugs (anti-mPEG end). In conclusion, the “double-attack” strategy of CD20 Ab-mPEG scFv and PLD provides an effective way to treat liquid tumors with the following advantages: (1) Non-chemical conjugation: the CD20 Ab-mPEG scFv can confer the PLD with liquid tumor specificity by anti-PEG scFv of the BsAb and maintain antibody dependent cellular cytotoxicity (ADCC) and complement dependent cytotoxicity (CDC) effects. (2) Cellular internalization ability enhancement: After PLD binding, the BsAbs increase the internalization ability of PLD to enhance the cellular cytotoxicity compared to the naïve PLD. (3) Drug resistance issues: our strategy can combat drug resistance by flexibly replacing the therapeutic antibody or any kind of PEGylated nanocarrier in response to shedding of antigens or multi-drug resistance genes in liquid tumors. Moreover, the CD20 Ab-mPEG scFv retains the Fc domain which can cause antibody-dependent cellular cytotoxicity (ADCC) and complement dependent cytotoxicity (CDC) to improve the cytotoxicity against Raji cells. Our CD20 Ab-mPEG scFv provides an effective double attack strategy against liquid tumors and may become second generation antibody drugs.

## Conclusions

Enhancement of tumor-targeting and cellular internalization can improve the therapeutic efficacy of liposomal drugs toward liquid cancer cells. We developed a humanized bispecific antibody (CD20 Ab-mPEG scFv) which retains the anti-CD20 targeting and therapeutic function, whilst simultaneously attracting PEGylated liposomal doxorubicin (PLD) to enhance the internalization and anticancer activity of PLD against liquid cancer cells. This “double-attack” strategy, using CD20 Ab-mPEG scFv and PLD, enhanced liquid-tumor therapeutic efficacy under non-chemical conjugation and maintained antibody dependent cellular cytotoxicity (ADCC) and complement dependent cytotoxicity (CDC) effects. Moreover, shedding of antigens or multi drug resistance issues related with liquid tumors can be addressed by flexibly replacing the therapeutic antibody or any kind of PEGylated chemotherapy drugs by using this strategy. We believe this double-attack strategy, combining CD20 Ab-mPEG scFv and PEGylated nanoparticles, can be applied in the clinic to treat liquid tumors and become next generation antibody drugs, and a novel therapeutic therapy.

## Materials and methods

### Cells and animals

Expi293F cells (Thermo Fisher Scientific) were cultured in Expi293 expression medium (Thermo Fisher Scientific) on shakers (25 mm shaking diameter) with a shake speed of 120 rpm in a humidified atmosphere of 8% CO_2_ in air at 37 °C. Raji cells were a kind gift from Dr. Dr. Steve R. Roffler (Institute of Biomedical Sciences, Academia Sinica, Taiwan) and cultured in RPMI1640 (Sigma-Aldrich) supplemented with 6 g/L HEPES, 2 g/L NaHCO_3_, 10% heat-inactivated BCS (HyClone), penicillin (100 U/mL), and streptomycin (100 mg/mL) at 37 °C in a humidified atmosphere of 5% CO_2_ in air. No authentication besides confirming surface expression levels of CD20 was performed by the authors. Healthy 3- to 6-week-old female BALB/c mice and SCID mice (C.B17/Icr-*Prkdc*^*scid*^/CrlNarl) were purchased from the National Laboratory Animal Center, Taipei, Taiwan. All animal procedures were performed in accordance with the Guidelines for Care and Use of Laboratory Animals of Kaohsiung Medical University and approved by the Institutional Animal Care and Use Committee (IACUC) of Kaohsiung Medical University (IACUC number: 105169).

### Construction and expression of bispecific antibodies and nanoparticles

Human BsAbs were created by linking the C-terminus of an anti-mPEG scFv (clone h15-2b) [11] or anti-dansyl (anti-DNS) scFv to an anti-CD20 antibody (Ofatumumab, clone: 2F2) via a flexible peptide (GGGGS)_3_ to form CD20 Ab-mPEG scFv and CD20 Ab-DNS scFv, respectively. The anti-DNS scFv has been described previously [43]. The VL-Cκ and VH-CH1-CH2-CH3-linker-scFv domains were separated with an IRES in the pLNCX retroviral vector (BD Biosciences, San Diego, CA) in the unique Hind III and Cla I restriction enzyme sites to generate pLNCX- CD20 Ab-mPEG scFv and pLNCX- CD20 Ab-DNS scFv plasmids. Each plasmid was scaled up by transformation into TOP10 *E*. *coli*, mixed with 100 ml Luria broth in a 250 ml baffled flask and shaken O/N at 220 r.p.m. Large-scale plasmid purifications were conducted using the PureLink HiPure Plasmid Midiprep Kit (Thermo Fisher Scientific) according to the manufacturer’s instructions. For protein production, plasmids were transfected into Epxi293F cells using ExpiFectamine and protocols provided by the manufacturer (Thermo Fisher Scientific). Transfected cells were grown at 37 °C in an 8% CO_2_ incubator while shaking at 125 r.p.m. for 5 days. Secreted protein was harvested by centrifugation at 1500 r.p.m. for 5 min. Supernatants were passed through 0.22 µm filters for purification. Purifications were conducted by directly incubating 250 ml transfected supernatant with 3 ml PBS-washed Protein G beads (Millipore). Beads were washed twice with PBS and once with PBS diluted tenfold. Protein was eluted from the beads by adding 3 ml 0.01 M citric acid, pH 3.0. After harvesting, the eluents were immediately neutralized by adding 1 ml 0.1 M Tris, pH 9.0. Protein molecular weight was found by SDS-PAGE. PEGylated liposomal doxorubicin (PLD) was purchased the clinical Lipo-Dox® (2 mg/ml pegylated liposomal doxorubicin HCl) from TTY Biopharm Co; particle size of Lipo-Dox, 98.8 ± 0.6 nm; polydispersity index value, around 0.2; zeta potential, − 43.4 ± 0.6 mA, as described in our previous study [11]. PEGylated Lipo-DiD was purchased DiD-labeled mPEGylated liposome from FormuMax.

### Bi-specific function of the CD20 Ab-mPEG scFv by flow cytometry

The anti-CD20 and anti-PEG ability of the BsAbs were determined by incubating 2 × 10^5^ Raji with 35 nM (200 μl) BsAbs for 1 h on ice. Unbound antibodies were removed by extensive washing in cold PBS containing 0.05% (wt/vol) BSA, followed by the addition of 1 μM (200 μl) PEGylated Lipo-DiD for 1 h on ice. After removal of unbound PEGylated Lipo-DiD by extensive washing in cold PBS containing 0.05% (wt/vol) BSA, the surface fluorescence of viable cells was measured on a FACScan flow cytometer (Merck Flow Cytometer, Guava easyCyte System).

### Internalization ability of PEGylated liposomal DiD mediated by CD20 Ab-mPEG scFv by flow cytometry

The internalization of the BsAbs was determined by incubating 5 × 10^5^ Raji with 35 nM BsAbs for 30 min on ice and then the unbound antibodies were removed by extensive washing in cold PBS containing 0.05% (wt/vol) BSA. The cells were treated with 2.5 μM PEGylated Lipo-DiD (Thermo Fisher Scientific) for 1 h on ice. After removal of unbound probes by extensive washing in cold PBS containing 0.05% (wt/vol) BSA, the cells were transferred to fresh culture medium and incubated for 6, 12, 24 h at 37 °C. Control cells were incubated at 4 °C for 24 h. PEGylated Lipo-DiD on the surface of Raji cells was determined by staining with 10 μg ml^−1^ 6–3 anti-PEG antibody for 30 min and 4 μg ml^−1^ goat anti-mouse IgG Fcγ-FITC (Jackson ImmunoResearch Laboratories). After extensive washing with PBS, the fluorescence of cells was measured with a Cytomics FC500 flow cytometer. Internalization rate is expressed as percentage luminescence of goat anti-mouse IgG Fcγ-FITC according to the following formula: percentage (%) of Q3/(Q2 + Q3).

### In vitro cytotoxicity of CD20 Ab-mPEG scFv combined with PLD

Raji cell lines (4 × 10^3^/well) were incubated with CD20 Ab-mPEG scFv or CD20 Ab-DNS scFv at 20 nM at RT for 20 min in a 96-well U-bottom plate (Nalge Nunc International, Roskilde, Denmark). PEGylated liposomal doxorubicin (PLD, TTY Biopharm Co.) was then added to the plate at the following final concentration: 50, 10, 2, 0.4 and 0.08 μg mL^−1^ for 3 h and the supernatant was replaced with 100 μL RPMI1640 medium containing 10% heat-inactivated bovine calf serum (BCS) and antibiotics (100 U/ml penicillin and 100 μg/ml streptomycin). Cell viability was measured with ATPlite Luminescence Assay System (Perkin-Elmer, Waltham, MA, USA) after 72 h of incubation. Results are expressed as percentage cell viability of luminescence as compared with untreated cells according to the following formula: percentage (%) cell viability = 100 × (treated luminescence/untreated luminescence). Data are presented as the mean of three independent experiments. The half maximal effective concentration (LC_50_) values were calculated by fitting the data to a log (inhibitor) versus response (variable slope model) with GraphPad Prism 6.0 (GraphPad Software, San Diego, CA, USA).

### Half-life of CD20 Ab-mPEG scFv in BALB/c mice

CD20 Ab-mPEG scFv (1 mg kg^−1^) or CD20 Ab-DNS scFv (1 mg kg^−1^) were injected intravenously (i.v.) into BALB/c mice (n = 3). Blood samples were collected via retro-orbital bleeds from each animal at the following time points: 30 min, 6 h, 12 h; and 1, 2, 3 and 7 d, and processed to collect serum. Sera collected were stored at approximately − 80 °C until analyzed by enzyme-linked immunosorbent assay (ELISA) for antibody concentrations. Ninety-six-well plates (Nalge Nunc International, Roskilde, Denmark) coated with 1 nM Goat anti-human IgG Fab for 2 h with 0.1 M NaHCO_3_ buffer, pH 9 at 37 °C. The plates were blocked overnight with PBS containing 5% (wt/olv) skim milk at 4 °C. The plates were washed three times with PBS with 0.02% (wt/vol) Tween20 (PBST) and one time with PBS. Plasma samples at dilution of 600-fold in 50 μL of PBS containing 2% (wt/vol) skim milk and graded concentrations of Herceptin as standard antibody were added to the plates for 1 h at room temperature (RT). The plates were washed three times with PBS with 0.02% Tween20 (PBST) and one time with PBS, followed by the addition of 0.4 μg mL^−1^ horseradish peroxidase (HRP)-conjugated goat anti-human IgG Fc antibody (Jackson Immunoresearch Laboratories, West Grove, PA, USA). The plates were washed again and bound peroxidase activity was measured by adding 150 μL/well ABTS solution [0.4 mg mL^−1^, 2,2′-Azino-bis-[3-ethylbenzothiazoline-6-sulfonic acid] (Sigma-Aldrich, St. Louis, MO, USA), 0.01% (v/v) H_2_O_2_, and 100 mM phosphate-citrate, pH4.0] for 1 h at RT. Color development was measured at 405 nm on a microplate reader (Molecular Devices, Menlo Park, CA, USA).

### Treatment efficacy of CD20 Ab-mPEG scFv combined with PLD in Raji-bearing mice

Mice were injected via the tail vein with 1 × 10^6^ Raji cells. One week later, the inoculated mice were divided into groups (n = 6 or 7) and administered via the tail vein with different group treatments (in 100 μL PBS) every other day. The treatments were: (1) PBS (100 μL), (2) CD20 Ab-mPEG scFv (1 mg kg^−1^), and (3) CD20 Ab-DNS scFv (1 mg kg^−1^). After 72 h, the mice were intravenously administrated PLD (2 mg kg^−1^). Raji cell progression was monitored weekly and the injections caused the mice to become paralyzed. Mice were sacrificed at the onset of paralysis or when the body weight dropped below 80% of the initial value; otherwise, the mice were maintained until 125 days and considered to be long-term survivors. Data was analyzed using GraphPad Prism 6.0. Survival was compared using Logrank test. Statistical significance was set at *P* < 0.05.

## Abbreviations

CD20: Cluster of differentiation 20
BsAb: Bispecific antibody
mPEG: Methyl polyethylene glycol
mAb: Monoclonal antibody
scFv: Single chain fragment variable
Fab: Antigen-binding fragment
HC-scFv: Heavy chain-scFv fragments
LC: Light chain
PLD: PEGylated liposomal doxorubicin
IC_50_: Half maximal inhibitory concentration
DNS: Dansyl
ALL: Acute lymphoblastic leukemia
CDC: Complement-dependent cytotoxicity
ADCC: Antibody-dependent cellular cytotoxicity
EPR: Enhanced permeation and retention
PBS: Phosphate buffered saline
PBST: Phosphate buffered saline w/Tween20
BSA: Bovine serum albumin
HRP: Horseradish peroxidase
RT: Room temperature
EGFR: Epidermal growth factor receptor
HER2: Human epidermal growth factor receptor 2
